# In Vitro Affinity Maturation of Nanobodies against Mpox Virus A29 Protein Based on Computer-Aided Design

**DOI:** 10.3390/molecules28196838

**Published:** 2023-09-28

**Authors:** Haiyang Yu, Guanchao Mao, Zhipeng Pei, Jinfeng Cen, Wenqi Meng, Yunqin Wang, Shanshan Zhang, Songling Li, Qingqiang Xu, Mingxue Sun, Kai Xiao

**Affiliations:** 1 School of Health Science and Engineering, University of Shanghai for Science and Technology, Shanghai 200093, China; 213332732@st.usst.edu.cn; 2 Lab of Toxicology and Pharmacology, Faculty of Naval Medicine, Naval Medical University, Shanghai 200433, China; guanchaomao@smmu.edu.cn (G.M.); pzpashin1206@smmu.edu.cn (Z.P.); cenjinfeng@139.com (J.C.); wenqimeng@smmu.edu.cn (W.M.); 15066916470@139.com (Y.W.); 18119965188@139.com (S.Z.); eddie886@yeah.net (S.L.); 3 Marine Biomedical Science and Technology Innovation Platform of Lingang Special Area, Shanghai 201306, China

**Keywords:** MPXV, A29 protein, nanobody, in vitro affinity maturation

## Abstract

Mpox virus (MPXV), the most pathogenic zoonotic orthopoxvirus, caused worldwide concern during the SARS-CoV-2 epidemic. Growing evidence suggests that the MPXV surface protein A29 could be a specific diagnostic marker for immunological detection. In this study, a fully synthetic phage display library was screened, revealing two nanobodies (A1 and H8) that specifically recognize A29. Subsequently, an in vitro affinity maturation strategy based on computer-aided design was proposed by building and docking the A29 and A1 three-dimensional structures. Ligand-receptor binding and molecular dynamics simulations were performed to predict binding modes and key residues. Three mutant antibodies were predicted using the platform, increasing the affinity by approximately 10-fold compared with the parental form. These results will facilitate the application of computers in antibody optimization and reduce the cost of antibody development; moreover, the predicted antibodies provide a reference for establishing an immunological response against MPXV.

## 1. Introduction

Mpox (MPX) is a zoonotic disease caused by the Mpox virus (MPXV), a member of the genus Orthopoxvirus (OPXV) in the family Poxviridae. Since 1 January 2022, 110 countries and regions have reported MPX cases to the World Health Organization (WHO). The WHO declared MPX a “Public Health Emergency of International Concern” on 23 July 2022 [1]. As of 17 March 2023, the WHO has reported 86,601 laboratory-confirmed cases and 1265 probable cases, including 112 deaths [2]. To date, the only means of preventing MPXV infections is the smallpox vaccination [1,3]. However, after 1980, vaccinating against smallpox was successively stopped in various countries, and >70% of the global population is exposed to the threat of MPXV. Therefore, since the eradication of smallpox, MPXV has become the most important pathogenic zoonotic orthopoxvirus in humans.

Two different forms of infectious virus particles develop during the replication–infection–transmission of MPXV: the mature virion (MV) and enveloped virion (EV) [4]. The MV, which is wrapped in >20 membrane proteins, is mainly responsible for transmission between hosts; the EV is primarily involved in cell-to-cell transmission [5]. The EV is formed after the MV passes through a cellular secretory transport system and acquires a second envelope, comprising eight unique proteins [6]. Among the proteins, A29, which is homologous to the vaccinia virus A27 protein, is one of the most widely studied targets [7]. However, most current serological assays are cross-reactive and cannot distinguish between individual species of OPXV because of the high protein homology. Alternatively, antibody-based immunological assays are ideal for diagnosis and monitoring. Therefore, specific monoclonal antibody studies against A29 are urgently required.

Nanobodies, used in a variety of viral, bacterial, and biomolecular assays, are promising alternatives for therapeutic and diagnostic purposes compared with traditional antibodies [8,9,10]. The low molecular weight of nanobodies (15 kDa) offers remarkable advantages [11,12,13], and highly variable complementary-determining regions (CDRs) allow nanobodies to recognize hidden epitopes more easily [14,15]. Fully synthetic phage display libraries combined with nanobodies are one of the most commonly used platforms for antibody detection. Any antibodies can be developed in theory [16,17,18]; however, the developed antibodies may have weak affinity [19] and require further affinity maturation. 

With technological advances in bioinformatics and computational biology, computer-aided antibody optimization and design have become widely used [20,21]. The cost and cycle time of rescreening secondary libraries [22,23,24] can be greatly reduced based on computer-aided optimization, which can achieve efficient mutations in a short time [25]. In particular, the effects of single- or multi-point amino acid site mutations can be predicted to guide the acquisition of affinity-enhancing antibodies (e.g., mSCM-AB2, Foldx, MutaBind2, BeAtMuSiC) [26,27,28,29,30]. 

In this study, we screened and identified two antibodies, A1 and H8, that specifically target the MPXV A29 protein using a previously established fully synthetic phage display library (the used library size was 1.44 × 10^10^) [31]. To further improve affinity for A1, antibody affinity maturation was performed in vitro using a computer-aided approach. MutaBind2 [29] combined with the mCSM-AB2 [27] platform was used to virtually screen affinity-improving mutants. Ultimately, three antibody mutants were designed and their affinities measured; the binding affinities were significantly improved by approximately 10-fold. This study confirms the feasibility of affinity maturation based on computer modeling and provides several high-affinity nanobodies against the MPXV A29 protein to promote the development of an efficient MPXV detection system. 

## 2. Results

### 2.1. Phage Screening of A29

**Figure 1:**
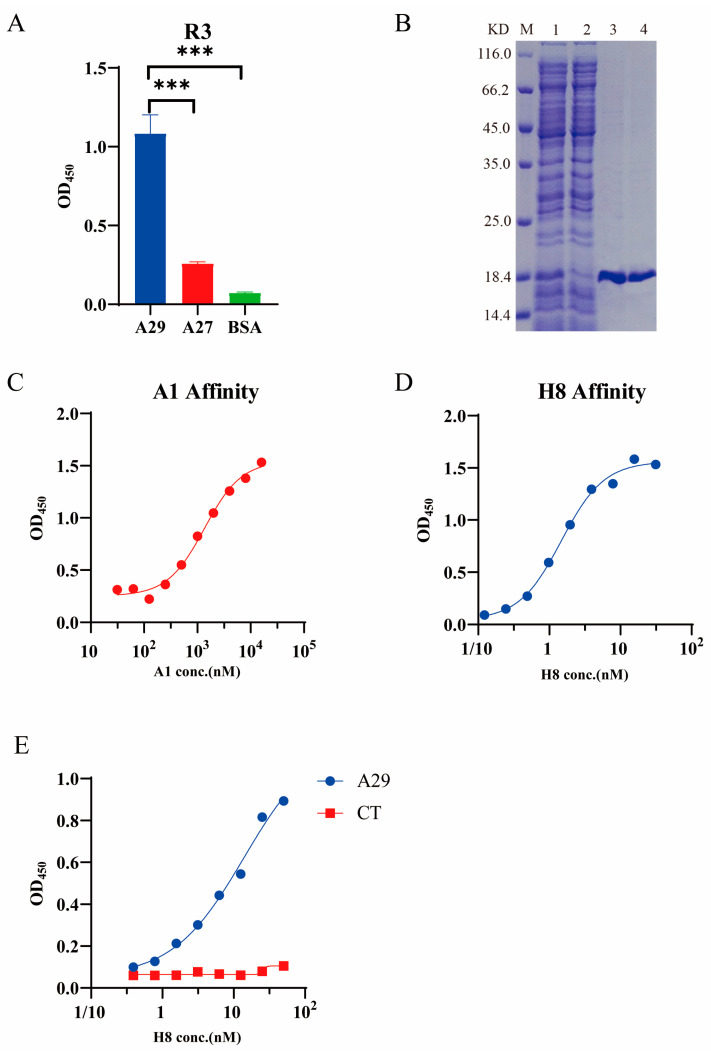
Initial anti-MPXV A29 protein nanobody preparation and validation. (**A**) Third round of phage supernatant screening using ELISA. A27 was used as a negative control for characterization of specificity (*n* = 3). *** *p* ≤ 0.001. (**B**) Sodium dodecyl sulfate-polyacrylamide gel electrophoresis (SDS–PAGE) analysis of purified initial antibodies. Lane M: molecular weight marker. Lanes 1 and 2: supernatant after ultrasonic crushing. Lane 3: A1. Lane 4: H8. (**C**,**D**) Antibody binding affinities (A1 and H8) to A29 (EC50_A1_ = 1.3 μM and EC50_H8_ = 1.5 nM). (**E**) Indirect ELISA of A1 and H8 targeting A29.

Three rounds of biological screening were performed to successfully enrich phages against A29. The panning strategy is displayed in the Supplemental Information. The vaccinia virus A27 protein [7] was used as a negative control during the screening process to improve antibody specificity. The phage titer results are shown in Figure S1. ELISA results indicated that the supernatant obtained after three rounds of elution could bind to the MPXV A29 protein with good specificity (Figure 1A). Then, we randomly selected 96 monoclonal phages from the supernatant for testing, from which 31 specific nanobodies were selected for sequencing. Two intact sequences, A1 and H8, were obtained after sequence analysis.

### 2.2. Antibody Expression, Purification, and Affinity Validation

PET-25b(+) prokaryotic expression vectors (Figure S2) were successfully constructed for the nanobodies A1 and H8 and induced in *E. coli* BL21 receptor cells. After purification, the molecular weight of the protein was consistent with the theoretical molecular weight as determined by SDS–PAGE (Figure 1B). Affinity analyses of the antibodies were conducted using ELISA, and the half maximal effective concentration (EC50) of A1 and H8 against A29 were approximately 1.3 and 1.5 nM, respectively (Figure 1C,D). A1 had a lower affinity and needed to be further optimized in vitro.

In addition, indirect sandwich ELISA was conducted to determine whether the two antibodies had overlapping epitopes. The results showed that the optical density (OD) decreased as the concentration of H8 increased; A1 and H8 had different antigen recognition sites (Figure 1E). 

### 2.3. Computer-Aided Modeling and Docking of Structures

**Figure 2:**
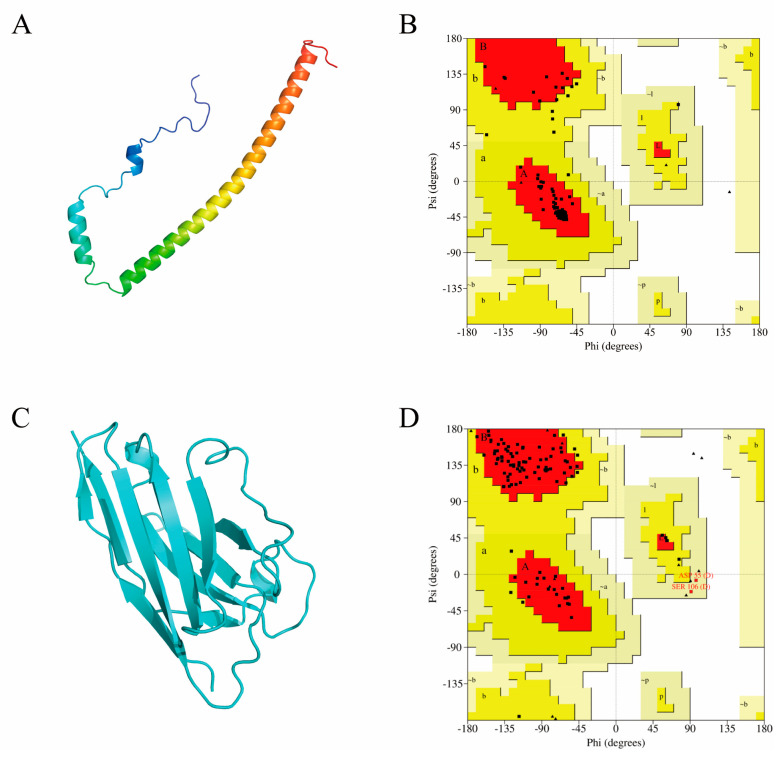
Three-dimensional structures and evaluation of antigens and antibodies. (**A**) MPXV A29 protein scratch modeling three-dimensional model. (**B**) Ramachandran plot of A29. Red, fully allowed region; yellow, permissive region; blank, non-permissive region. (**C**) Three-dimensional structure obtained from homology modeling of the antibody A1. (**D**) Ramachandran plot of the A1 nanobody.

A computer model of A1 was constructed using the Swiss model [32] by selecting a homologous antibody (PDB:7eow) with 84.68% sequence similarity as the best template. The crystal structure of A29 was previously unknown; therefore, the model was built using the AlphaFold 2 2.3.2 program [33]. As a result, the complete three-dimensional structures were obtained for subsequent molecular recognition studies (Figure 2A,C). No amino acid residues were located in the torsion angle-forbidden region of the Ramachandran plots (Figure 2B,D). Consequently, the dihedral angles of all amino acid residues of the modeled A1 and A29 proteins were within a reasonable range, which conformed to the stereochemical energy rules.

**Figure 3:**
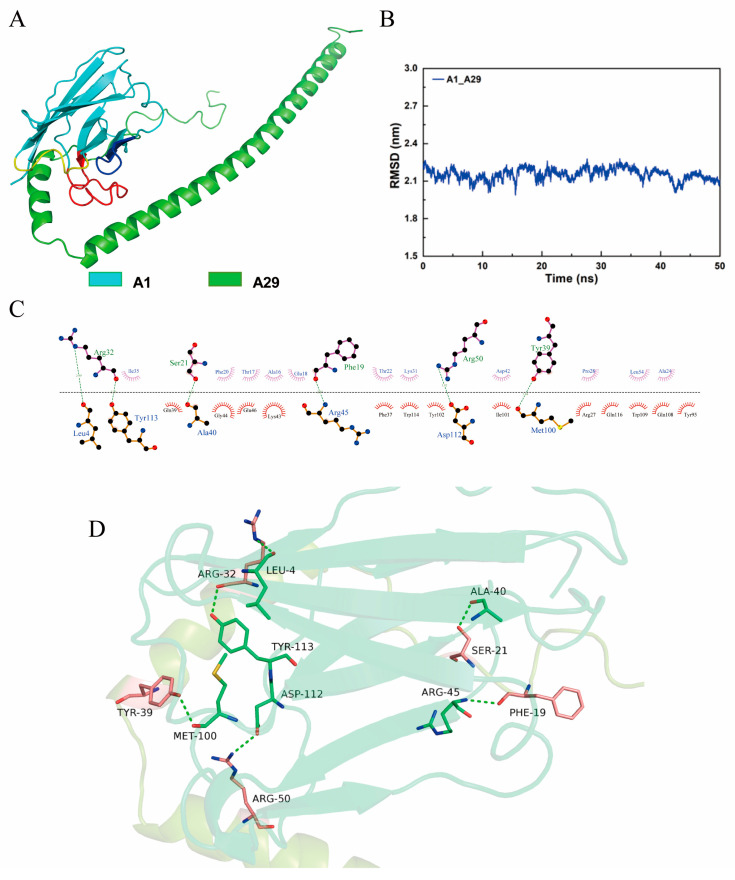
Analysis of antibody–antigen complex structures and interactions. (**A**) Three-dimensional complex structure of A1 antibody (cyan) docked with A29 protein (green). CDR1, CDR2, and CDR3 are shown in yellow, blue, and red, respectively. (**B**) Root mean square deviation (RMSD) calculation during the 50 ns MD simulation of the A1 nanobody and A29 protein. (**C**) Diagram of the interaction between A1 and A29 in two dimensions. Hydrophobic interactions are represented by dentate amino acids and hydrogen bonding interactions are represented by green dashed lines. (**D**) PyMOL visualization of A1 (green) and A29 (pink) three-dimensional structural interactions. Hydrogen bonding interactions are represented by green dashed lines.

To investigate the binding regions and interaction patterns between the A1 and A29 proteins, the professional HDCOK program for protein–protein docking was used to predict the composite structure, and the structure with the best docking score was selected as the standard for subsequent interaction analysis [34,35]. However, this score cannot be directly considered as the true value of the affinity for binding between two molecules because there are no experimental data for correction. The best docking result had a docking score of −279.89 and a confidence score of 0.931, indicating that the docked complex model had a high confidence level. The optimal docking model exhibited binding of the bottom of the β-sheet domain on A1 to the gap formed by the bending of the two segments of the A29 molecule, resulting in a more stable interaction between the two molecules (Figure 3A). The interaction patterns of the binding regions of the A1 and A29 proteins were then analyzed. A total of six sets of hydrogen bond interactions and numerous hydrophobic interactions were formed, which improved binding of the A1 and A29 proteins (Figure 3C,D). 

To gain a deeper overview of the stability of the A1–A29 protein complex structure, a 50 ns molecular dynamics (MD) simulation was performed using the Gromacs 2018.4 program [36]. RMSD of all atoms in the A1–A29 system did not show significant differences or large fluctuations throughout the simulation, indicating that there were no significant conformational changes in the docked complexes. The MD simulations were stable and reliable, based on force field parameters, and could be used for in-depth analysis (Figure 3B).

### 2.4. Key Residue Positions for Affinity Maturation

The identification of key residues for A1–A29 protein interactions is essential. Mutagenesis of CDR is helpful for increasing affinity, as it allows sufficient diversity to be obtained without disrupting the overall structure of the nanobody [37]. The interfacial residues in the CDRs of the A1–A29 complex structure were statistically analyzed using InterProSurf and PyMOL, and nine active residues located on A1 were identified: R27, M100, I101, Y102, Q108, W109, S110, D112, and Y113 (Table 1).

### 2.5. Mutagenesis for Affinity Maturation In Silico

Computational prediction is the most critical step in computer-aided affinity maturation. Three prediction rounds were performed using the MutaBind2 and mCSM-AB2 platform. This combined strategy narrowed the scope of mutant screening and improved the accuracy of the prediction results. The results of both servers were calculated as the change in binding free energy (ΔΔG; kcal/mol). Generally, a lower free energy results in a more stable complex structure and increased affinity, whereas a higher free energy results in decreased affinity.

Considering the specificity of cysteine and alanine, the nine interfacial residues were mutated to 18 other natural amino acids (except cysteine and alanine) in the first round of calculations, and 162 single point mutations were obtained. After the first round of the joint strategy, the affinities of 10 single mutants for five amino acid sites (R27, M100, I101, Q108, and S110) were predicted to be enhanced by both servers (Table 2). Thereafter, 39 double-point mutants were obtained by combining the 10 mutants. In the second round of calculation, the multiple mutation module of MutaBind2 was used for multiple mutation prediction. Compared with the parental nanobody, the affinities of 32 double mutants out of 39 predictions were improved (ΔΔG < 0; Table 3). Before the third round of calculations, we sorted the 32 double mutants as described in the Methods section. Ultimately, the overall sorting process narrowed the selection from 32 increased affinity double mutants to 11 double mutants in the next prediction step (Table 3). In the third round, 11 triple mutants were obtained by combining 11 double mutants, all of which had an improved affinity for the parental nanobody (Table 4). By the standard of ΔΔG < −2, both triple mutants, M100E Q108Y I101N (M1) and R27Y Q108Y M100E (M2), were selected for experimental verification of affinity. Interestingly, in the process of selecting the best mutants, the double mutant in round two, R27Y Q108Y (M0), also presented a significant improvement over the other mutants; therefore, it was considered. 

### 2.6. Affinity and Recognition Site Validation after Mutation

**Figure 4:**
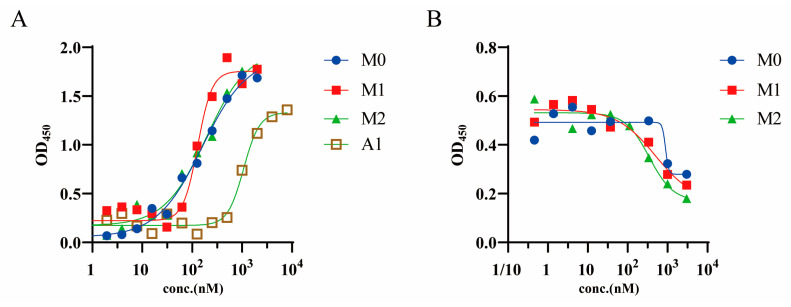
Analysis of affinity and recognition sites of mutants. (**A**) Binding activity of mutants and comparison with the initial antibody A1. (**B**) Confirmation of nanobody epitopes.

Overlap extension (OE)–PCR [38] was used to obtain three mutant genes and their corresponding proteins (Figure S3). The affinity of the mutants M0–M2 was analyzed using ELISA. After computerized optimization, the affinities of all mutants were significantly higher than that of the parental nanobody A1, and the EC50 values of the three mutants binding A29 were 159.1, 128.9, and 191.0 nM for M0–M2, respectively. Compared with A1, M1 had the best binding affinity (approximately 10-fold higher; Figure 4A), indicating that the virtual screening strategy was effective in obtaining improved antibody mutants.

Considering possible epitope drift after computer-aided design, we investigated whether the epitopes of A29 recognized by the three mutant antibodies were identical to those recognized by the parental nanobody. Competitive ELISA showed that the three optimized nanobodies were all able to effectively compete with A1 for binding to A29 (Figure 4B). Thus, the three mutant antibodies showed no significant epitope drift.

## 3. Discussion

MPXV was discovered in 1958, first reported in humans in 1970, and attracted widespread attention in 2022 [39]. Although the spread of MPXV is limited by its mode of transmission, the lack of a proven specific antiviral drug and vaccine for MPX sustains the threat of MPXV [40]. Currently, detecting and preventing MPX remains a great challenge [41]. The high affinity antibody in this study has a great potential to detect A29 protein, the target of MPXV, and promote MPXV-related research. Meanwhile, with reference to the experience of the SARS-CoV-2, it lays the foundation for the development of kits or colloidal gold test strips for the rapid detection of MPXV. In addition, several studies have shown that, similar to homologous proteins, the tail structural domain of A29 contains a heparin-binding domain [42,43]. Although we have not yet verified the binding sites and neutralization effects of these antibodies, according to the three-dimensional complex structure obtained from the computer-aided design, A1 and the two mutants can bind sufficiently to the heparin-binding domain of A29. Therefore, it is possible that nanobodies have therapeutic effects on MPXV and provide a reference and research tool for future MPXV-related treatments.

**Figure 5:**
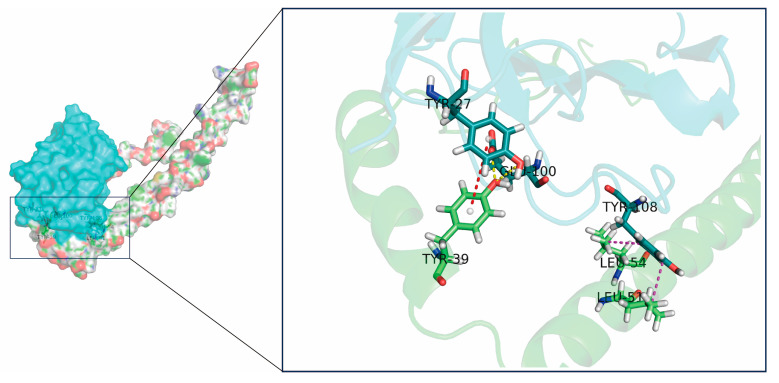
Mutation site analysis of the best mutant, M1. The three new amino acid sites of the mutant antibody M1 compared with the parental antibody are shown in cyan, and amino acid sites of the A29 protein and their interactions with mutated residues are represented in green. Hydrogen bonding interactions are represented by yellow dashed lines, hydrophobic interactions are represented by purple dashed lines, and aromatic–aromatic interactions are represented by red dashed lines.

Recently, artificial intelligence (AI) has become a popular topic in drug development. Computer-aided design has accelerated drug development and increased its success rate, in which homology modeling and molecular docking techniques can improve the efficiency of some large-molecule drugs (e.g., antibodies). In this study, a low-cost, timesaving programmed affinity maturation scheme for nanobodies was used as a reference for computer-aided antibody optimization. Although there may be biases in the computer algorithm, the computer values are generally logical and reasonable, and do not affect the selection of the best mutant. Understanding the structural basis of affinity improvement was helpful for the computer affinity maturation algorithm. Additional structural and sequence-based information can be captured using mCSM-AB2, which is trained on a large dataset that includes graph-based features, interatomic interactions, and evolutionary and energetic features. Therefore, a more accurate prediction of antibody-to-antigen changes upon mutation can be achieved [27]. Moreover, MutaBind2, which employs a minimization protocol and scoring function consisting of seven terms, can improve the accuracy of mutations [29]. In addition to individual mutations, it can predict the effect of multiple mutations on protein-binding affinity. Furthermore, mutations at multiple sites were also more effective, which is consistent with previous studies where mutations at multiple sites resulted in moderate increases in affinity compared with single-site mutations [44,45]. However, the effects of multi-point mutations on nanobodies are complex. Exploring the effects of multipoint mutations on antibody structures might facilitate the development of computer-aided affinity maturation in the future. In terms of the antibody-antigen complex structure, the key reason for the improved affinity of the mutants could be the incorporation of aromatic residues, which facilitates the stability of the protein structure [46,47,48]. The interaction between M1 and A29 was enhanced after mutations at three residues (Figure 5), and four new sets of hydrogen bonds were incorporated into the antibody–antigen interaction. At the mutation site, a new hydrogen bond was formed under the combined interaction of E100 at M1 and Y39 at A29, which contributed to the improvement in the binding affinity. In addition, Y108 of M1 was linked to L51 and L54 of A29 via new hydrophobic interactions. Notably, the existing hydrogen bond between R27 at A1 and Y39 at A29 before the mutation was not only maintained after the R27Y mutation, but a new aromatic–aromatic interaction also formed. There may be different mechanisms for improving binding affinity, even at the same sites. Importantly, the best mutant, M1, retained two aromatic residues based on the double mutant M0, with the addition of a new mutation site. Thus, aromatic residues may be important for improving antibody affinity. 

This study has some limitations. In the initial modeling, the antibody A1 model was obtained using a mature homology modeling approach with a high degree of confidence. However, the A29 model has no defined crystal mechanism, and therefore modeling accuracy may be in doubt. In addition, the choice of complexes after docking of antibody antigens also has different possibilities owing to many different conformations. Moreover, affinity validation differences may exist between experimentally determined results and computer values, probably owing to uncertainty of the computer algorithm. However, overall, the computer values were logical and plausible, and this did not affect the selection of the best mutant.

## 4. Materials and Methods

### 4.1. Sequencing

Sequencing was performed by Sangon Biotech Inc. (Shanghai, China).

### 4.2. Phage Screening for Nanobody Selection

Three rounds of gradient encapsulation were performed using different concentrations (10, 5, and 1 μg/mL) of the MPXV protein A29 (Sino Biological Inc., Beijing, China). The A29 protein and recombinant vaccinia virus protein A27 (CUSABIO, Inc., Wuhan, China) were diluted with phosphate-buffered saline (PBS) and encapsulated in well plates at 4 °C overnight. After washing thrice with 0.1% PBS with Tween 20 (PBST), the plates were blocked with 5% skim milk at 25 °C for 2 h. After three subsequent washes, the pretreated phage library was added to the A27 protein wells and incubated at 25 °C for 1 h. The library liquid from the A27 wells was then transferred to the A29 wells and incubated for 1 h. After washing the unbound phages with PBST, Gly-HCl (pH 2.2) was added to the wells and neutralized. Finally, the phage titer in the eluate was detected and amplified. 

### 4.3. Phage Enzyme-Linked Immunosorbent Assay (ELISA)

First, 100 μL/well of 2 μg/mL A29 protein, 2 μg/mL A27 protein, and 0.5% bovine serum albumin (BSA) were added to well plates and incubated at 4 °C overnight. Thereafter, 5% skim milk was added for 2 h at 25 °C, before 100 μL phage clone supernatant was added to each well and incubated at 25 °C for 1 h. Next, 100 μL/well of secondary anti-M13 antibody (HRP; Sino Biological Inc.) was added and incubated for 1 h at 25 °C. Tetramethylbenzidine (TMB) color development solution (Beyotime Inc., Shanghai, China) was used for color rendering, and 2 M H_2_SO_4_ was added to terminate the reaction. Absorbance was measured at 450 nm to assess the specificity of the supernatant.

### 4.4. Construction, Expression, and Purification of the Nanobody

Nanobody fragments were amplified and recombinantly ligated using polymerase chain reaction (PCR). Next, the recombinant product was mixed with DH5α receptor cells for 30 min and then heat-stimulated at 42 °C for 90 s. The cells were coated on culture plates at 37 °C overnight. After extracting the plasmid and transforming it into *E. coli* BL21 cells using the same method, expression was induced by adding 1 mM IPTG at 220 rpm and 20 °C and mixing overnight. The bacterial broth was then sonicated for 20 min under 1 KW ultrasonics for 5 s with intervals of 5 s. The nanobody supernatant was obtained by centrifugation and purified by Ni^2+^ affinity chromatography. The antibody proteins were washed and eluted with 10 and 250 mM imidazole, respectively. Finally, protein purity was detected using sodium dodecyl sulfate-polyacrylamide gel electrophoresis (SDS-PAGE). 

### 4.5. Affinity ELISA

A mixture of 100 μL/well of 2 μg/mL A29 protein, 2 μg/mL A27 protein, and 0.5% BSA was added to wells and incubated at 4 °C overnight. Next, 5% skim milk was added and incubated at 25 °C for 2 h. Thereafter, 100 μL antibodies obtained from a two-fold ratio gradient dilution was added to each well and incubated at 25 °C for 1 h. A secondary anti-HA tag antibody (HRP; Sino Biological Inc., Beijing, China) was added and incubated at 25 °C for 1 h. TMB solution was used for color rendering, and 2 M H_2_SO_4_ was added to terminate the reaction. The absorbance was measured at 450 nm and a binding curve was constructed to analyze antibody affinity.

### 4.6. Antigen Epitope Analysis

Wells were filled with 100 μL/well of 2 μg/mL A1 antibody and incubated at 4 °C overnight. Next, 8 μg/mL A29 protein was added and incubated at 25 °C for 1 h. After washing with PBST, H8 antibody with an HA label was added to the wells after gradient dilution for 1 h. Finally, anti-HA antibody (HRP) was added and incubated at 25 °C for 1 h. TMB solution was used for color rendering, and the reaction was terminated by adding 2 M H_2_SO_4_. Absorbance was measured at 450 nm and a curve was constructed to analyze the antigen-binding epitopes of the antibody.

### 4.7. Homology Modeling

The amino acid sequence of A29 was obtained from the Uniprot website (https://www.uniprot.org/, accessed on 12 March 2023) and then uploaded to AlphaFold [33]. The monomeric structure was selected for ab initio modeling operations, and the model with the highest rank value was used for subsequent studies according to the results. In addition, the amino acid sequence of the A1 antibody was obtained based on the sequencing results and uploaded to the Swiss-model [32] homology modeling online server (https://swissmodel.expasy.org/, accessed on 14 March 2023) to construct the three-dimensional theoretical structure of the antibody. Eight templates were selected based on sequence similarity. Subsequently, computational modeling was performed using these templates, and eight A1 models with different conformations were obtained. As the score assigned by the modeling server did not provide the best model accuracy, we used SAVES 6.0 (https://saves.mbi.ucla.edu/, accessed on 14 March 2023) for further evaluation and optimization. After uploading the eight models, the best A1 model, model07 (template PDB:7eow), with the highest overall quality factor of 99.1304, was selected based on the overall ERRAT evaluation score. A Ramachandran plot was used to illustrate the bond rotation between the α-carbon atom and the carbonyl carbon atom within the peptide bond versus the bond rotation between the α-carbon atom and the nitrogen atom in the stereo structure of a protein or peptide. This technique is mainly used to indicate allowed and unallowed conformations of amino acid residues in a protein or peptide. The Ramachandran plot is divided into three main regions: the fully allowed (red), permissive (yellow), and non-permissive (blank) regions.

### 4.8. Molecular Docking

A professional HDCOK program [34,35] for protein–protein docking was used to predict the structure of the A1–A29 complex, using antibodies as receptors and antigens as ligands for molecular docking. Docking scores were calculated based on the ITScorePP or ITScorePR iterative scoring functions. A more negative docking score indicates that the structural model is likely to have greater binding and stronger interactions. Considering that the docking scores for protein–protein complexes in PDB are typically approximately −200 or better, we empirically defined a docking score-dependent confidence score to indicate the likelihood of the binding of two molecules as follows: confidence score = 1.0/[1.0 × 10^2^ × (Docking_Score + 150)].

When the confidence score was >0.7, the two molecules were highly likely to combine; when the confidence score was 0.5–0.7, the two molecules were likely to combine; when the confidence score was <0.5, the two molecules were unlikely to combine. Finally, the structure with the best docking score was selected as the standard for subsequent interaction analysis.

### 4.9. MD Simulation

The root mean square deviation (RMSD) is an important measure of the stability of the system and represents the sum of all atomic deviations of the conformation at a given instant from the target conformation. All MD simulations and their analyses were performed using the Gromacs 2018.4 program [36], applying the Amber14SB all-atom force field [49] and TIP3P water model [50]. During MD simulations, all bonds involving hydrogen atoms were constrained using the LINCS algorithm [51] with an integration step of 2 fs. The electrostatic interactions were calculated using the particle mesh Ewald (PME) method. The nonbonded interaction truncation value was set to 10 Å and updated every 10 steps. The simulation temperature was maintained at 298.15 K using the V-rescale temperature coupling method, and the pressure was controlled at 1 bar using the Parrinello–Rahman method [52]. First, the steepest descent method was used to minimize the energy of the two systems to eliminate too close contact between atoms; subsequently, the 1 ns NVT and NPT equilibrium simulations were performed at 298.15 K. Finally, 50 ns MD simulations were performed on the system, with conformations saved every 10 ps. Simulation results were visualized using the Gromacs-Embedded 2018.4 program and VMD 1.9.3. 

### 4.10. Identifying Key Residue Positions

The CDR region of the A1 antibody was obtained using the AbRSA [53] server (http://aligncdr.labshare.cn/aligncdr/cdrs.php, accessed on 25 March 2023). The InterProSurf [54] online server (https://curie.utmb.edu/usercomplex.html, accessed on 26 March 2023) was used to analyze and predict amino acid residues of the A1 antibody located at the interface of the interaction with A29. In addition, to avoid missing residues and to improve accuracy and reliability, PyMOL (http://www.pymol.org/, accessed on 16 March 2023) was used for the statistical analysis of the interface residues of A1 antibodies within 5 Å of A29. 

### 4.11. In Silico Affinity Maturation

MutaBind2, combined with the mCSM-AB2 platform, was used to optimize the affinity of A1. The results were all changes in binding free energy (ΔΔG; kcal/mol); reduced free energy indicated a more stable complex structure and increased affinity, and the opposite was observed with decreased affinity. However, MutaBind2 is the mutant minus the free energy change of the wild type (ΔΔG_MutaBind2_ = ΔG_mut_ − ΔG_wt_), while the opposite is true for mCSM-AB2 (ΔΔG_mCSM-AB2_ = ΔG_wt_ − ΔG_mut_). Therefore, for the calculated results, MutaBind2 was chosen to have negative values and mCSM-AB2 was chosen to have positive values.

Nine residue sites were changed to 18 different natural amino acids in the first round of calculations for a total of 162 single point mutations, and 10 single mutants were selected for both servers to predict affinity enhancement. In the last two rounds of prediction, the results were deleted to reduce workload and error. The binning process was based on residue type, locus diversity, and visual inspection. First, considering that almost all ΔΔG values and means in the first round of MutaBind2 calculations were between −1 and 0, double mutant sites with lower ΔΔG (ΔΔG < −1) in the second round of calculations compared with the first round were selected. In addition, the 15 double mutants with ΔΔG < −1 were further sorted by selecting the double mutant with an absolute value of ΔΔG greater than the sum of its two single mutations (e.g., R27F Q108W: |ΔΔG|_R27F Q108W_ > |ΔΔG|_R27F_ + |ΔΔG|_Q108W_). The round 2 sorting method was then used. Two triple mutations with ΔΔG < −2 were selected in the third round.

### 4.12. Quantification and Statistical Analysis

InterProSurf and PyMOL 1.8.x were used for statistical analysis of interface residues. Statistical significance was defined as *p* < 0.05. 

## 5. Conclusions

In conclusion, two parental nanobodies against the MPXV A29 protein were obtained from a fully synthetic phage display library, followed by computer-assisted prediction of the three antibodies. Compared with the parental nanobody A1, a significantly higher affinity and no significant drift in the recognition epitopes were observed. This study draws attention to MPX, an increasingly present zoonotic disease, and provides an avenue for early diagnosis and treatment. Furthermore, the study demonstrates the feasibility of computer-aided design in antibody optimization, which also contributes to antibody development and cost reduction. Development of AI technologies in the future will further improve the accuracy and applications of computer-aided drug design.

## Figures and Tables

**Table 1:** Binding site analysis of A1 nanobody CDRs using two criteria.

Interface Residues Analysis		PyMOL Analysis	
1–5	-	1–5	-
27	ARG(R)	27	ARG(R)
37	PHE(F)	37–46	-
39	GLN(Q)	95	TYR(Y)
42–46	-	99	ALA(A)
95	TYR(Y)	100	MET(M)
100	MET(M)	101	ILE(I)
101	ILE(I)	102	TYR(Y)
102	TYR(Y)	108	GLN(Q)
108	GLN(Q)	109	TRP(W)
109	TRP(W)	110	SER(S)
110	SER(S)	112–116	-
112–116	-	117	GLY(G)
124	SER(S)	124	SER(S)

The selection of overlapping residues as the site of mutation. Residue positions are on the left and corresponding residue names are on the right.

**Table 2:** First round ΔΔG of virtual single point mutation prediction by the joint platform.

Mutants	MutaBind2	mCSM-AB2
R27F	−0.86	0.74
R27Y	−1.07	0.45
M100E	−0.39	1.07
M100D	−0.18	0.51
I101N	−0.03	0.61
I101S	−0.37	0.14
Q108F	−0.72	0.21
Q108W	−0.41	0.49
Q108Y	−0.7	0.71
S110Y	−0.23	1.34

ΔΔG changes indicate an increase in affinity used for the next round of predictions. Increased affinities are shown as negative values for MutaBind2 but positive values for mCSM-AB2.

**Table 3:** Second round ΔΔG of double point mutations by the MutaBind2.

Double Mutants	MutaBind2	Double Mutants	MutaBind2
R27F M100E	−0.37	M100E Q108F	−0.91
R27F M100D	−0.2	M100E Q108W	−0.38
R27F I101N	−0.59	**M100E Q108Y**	**−1.21**
R27F I101S	−0.48	M100D Q108F	−0.64
R27F Q108F	−1.44	M100D Q108Y	−0.68
**R27F Q108W**	**−1.51**	**I101N Q108F**	**−1.58**
R27F Q108Y	−1.5	I101N Q108W	−0.54
R27F S110Y	−1.05	**I101N Q108Y**	**−1.45**
R27Y M100E	−0.53	**I101N S110Y**	**−1.07**
R27Y M100D	−0.14	**I101S Q108F**	**−1.51**
R27Y I101N	−0.34	**I101S Q108W**	**−1.01**
R27Y I101S	−0.24	**I101S Q108Y**	**−1.76**
R27Y Q108F	−1.41	I101S S110Y	−0.52
**R27Y Q108W**	**−1.74**	Q108F S110Y	−0.94
**R27Y Q108Y**	**−2.11**	**Q108W S110Y**	**−1.27**
R27Y S110Y	−0.95	Q108Y S110Y	−0.95

Bold denotes double mutations sorted for the next prediction step. R27Y Q108Y was the final mutant used for validation.

**Table 4:** Third round ΔΔG of triple mutations by the MutaBind2.

Triple Mutants	MutaBind2
R27F Q108W I101S	−1.77
R27F Q108W S110Y	−1.66
R27Y I101S Q108W	−0.84
R27Y Q108W S110Y	−1.87
**R27Y M100E Q108Y**	**−2.31**
R27Y I101N Q108Y	−1.79
R27Y I101S Q108Y	−1.57
**M100E I101N Q108Y**	**−2.02**
M100E I101S Q108Y	−1.98
I101N Q108W S110Y	−0.89
I101S Q108W S110Y	−0.83

Bold denotes mutants used for experimental validation.

## Data Availability

Data generated or analyzed in this study are included in this published article and its Supplementary Information Files. Further queries should be directed to the corresponding author.

## Supplementary Materials

The following supporting information can be downloaded at: https://www.mdpi.com/article/10.3390/molecules28196838/s1, Table S1. Panning strategy for screening specific antibodies against the MPXV A29 protein. Figure S1. Phage supernatant titer assay in triple screening; Figure S2. Construction of primary antibody expression plasmids. Figure S3. Expression and purification of mutants.
