# Supplementary material for: In Vitro Affinity Maturation of Nanobodies against Mpox Virus A29 Protein Based on Computer-Aided Design
Source: Molecules. 2023 Sep 28;28(19):6838. doi: 10.3390/molecules28196838 (PMC10574621; doi:10.3390/molecules28196838)
Supplement: Supplementary file 1 [file molecules-28-06838-s001.zip › molecules-2597524-supplementary.pdf]

Supplementary Table S1. Panning strategy for screening specific antibodies against the MPXV A29 protein.

Round	Concentration (μg/mL)	Elution	Wash times	Input (pfu/mL)	Output (pfu/mL)	Recovery
1	10	Acid-base	20	2E14	5E6	2.5E-8
2	5	Acid-base	20	1E14	2E8	2E-6
3	1	Acid-base	30	6E13	3E7	5E-7

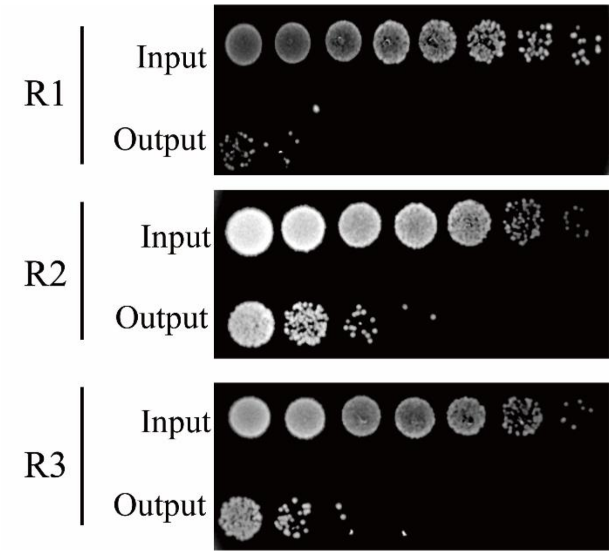

Supplementary Figure S1. Phage supernatant titer assay in triple screening.

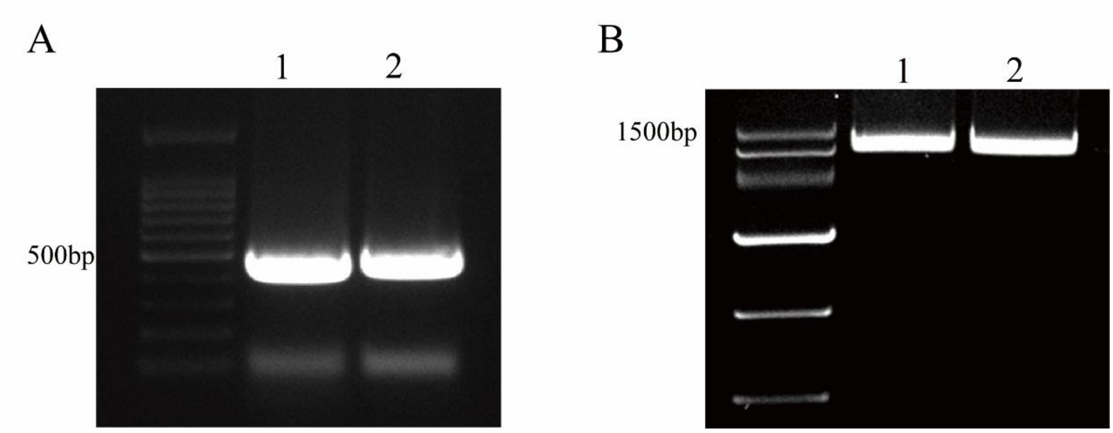

Supplementary Figure S2. Construction of primary antibody expression plasmids. (**A**) Acquisition of antibody A1 and H8 gene by PCR. Lane 1: A1. Lane 2: H8. (**B**) pET-25b(+) expression plasmid after digestion. Lane 1 and 2: pET-25b(+).

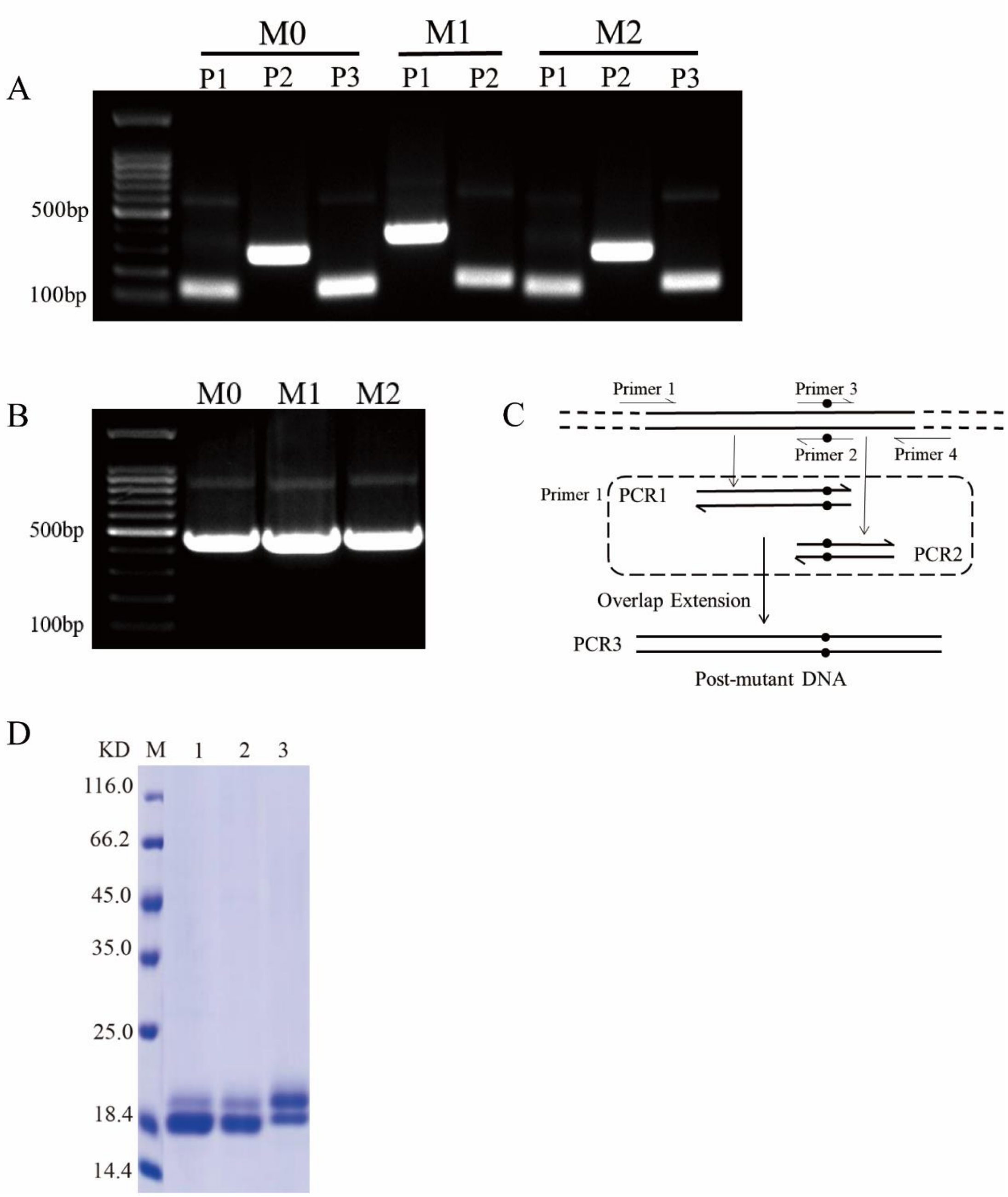

Supplementary Figure S3. Expression and purification of mutants. (**A**) Segmentation of target genes by designing primers to insert mutated genes at different points. Different lanes represent different fragments of each mutant. (**B**) Complete mutant gene obtained by linking the fragment with the mutant gene by PCR. (**C**) Theory of gene splicing by overlap extension PCR (SOE-PCR). (**D**) SDS-PAGE analysis for purified mutants. Lane M: molecular weight marker. Lane 1: M0. Lane 2: M1. Lane 3: M2.
